# Mitochondrial genome features and systematic evolution of *diospyros kaki* thunb 'Taishuu'

**DOI:** 10.1186/s12864-024-10199-0

**Published:** 2024-03-18

**Authors:** Yunliang Yang, Chao Duan

**Affiliations:** Cotton Research Institute of Shanxi Agricultural University, Yuncheng, 044000 China

**Keywords:** Mitochondrial genome, Persimmon, Repeat sequences, Evolutionary analysis

## Abstract

**Background:**

'Taishuu' has a crisp texture, abundant juice, and sweet flavor with hints of cantaloupe. The availability of mitochondrial genome data of *Diospyros* species is far from the known number of species.

**Results:**

The sequencing data were assembled into a closed circular mitochondrial chromosome with a 421,308 bp length and a 45.79% GC content. The mitochondrial genome comprised 40 protein-coding, 24 tRNA, and three rRNA genes. The most common codons for arginine (Arg), proline (Pro), glycine (Gly), tryptophan (Trp), valine (Val), alanine (Ala), and leucine (Leu) were AGA, CCA, GGA, UGG, GUA, GCA, and CUA, respectively. The start codon for cox1 and nad4L protein-coding genes was ACG (ATG), whereas the remaining protein-coding genes started with ATG. There are four types of stop codons: CGA, TAA, TAG, and TGA, with TAA being the most frequently used stop codon (45.24%). In the *D. kaki* Thunb. 'Taishuu' mitochondrial genome, a total of 645 repeat sequences were identified, including 125 SSRs, 7 tandem repeats, and 513 dispersed repeats. Collinearity analysis revealed a close relationship between *D. kaki* Thunb. 'Taishuu' and *Diospyros oleifera*, with conserved homologous gene fragments shared among these species in large regions of the mitochondrial genome. The protein-coding genes ccmB and nad4L were observed to undergo positive selection. Analysis of homologous sequences between chloroplasts and mitochondria identified 28 homologous segments, with a total length of 24,075 bp, accounting for 5.71% of the mitochondrial genome. These homologous segments contain 8 annotated genes, including 6 tRNA genes and 2 protein-coding genes (rrn18 and ccmC). There are 23 homologous genes between chloroplasts and nuclei. Mitochondria, chloroplasts, and nuclei share two homologous genes, which are trnV-GAC and trnW-CCA.

**Conclusion:**

In conclusion, a high-quality chromosome-level draft genome for *D. kaki* was generated in this study, which will contribute to further studies of major economic traits in the genus *Diospyros*.

**Supplementary Information:**

The online version contains supplementary material available at 10.1186/s12864-024-10199-0.

## Introduction

*Diospyros Linn* is a genus in the family *Ebenaceae* under the class of dicotyledonous plants. It comprises approximately 500 species worldwide and is widely distributed in tropical and subtropical regions [1]. *Diospyros kaki* Thunb., commonly known as persimmon, can be classified into two major categories based on the genetic traits linked to the natural de-astringency of its fruits: pollination constant & non-astringent (PCNA) and non-pollination constant & non-astringent (non-PCNA). Based on the regulation of different gene loci, PCNA can be further categorized into Japanese PCNA (JPCNA) and Chinese PCNA (CPCNA), whereas non-PCNA can be sub-classified into pollination-variant non-astringent (PVNA), pollination-constant astringent (PCA), and pollination-variant astringent (PVA). Persimmons are highly valued fruits due to their broad developmental prospects. They naturally lose astringency upon ripening, conferring excellent palatability. They possess high nutritional and health benefits compared to astringent persimmons [2, 3]. 'Taishuu' is a cultivar of PCNA persimmon developed in Japan [4]. It has a crisp texture, abundant juice, and sweet flavor with hints of cantaloupe, exhibiting superior quality to other persimmon varieties. After ripening, the fruit turns orange-red and gains an average weight of 200–400 g per fruit. The flesh is delicate and plump, with a long shelf life, facilitating effective storage and transportation (Fig. S1).

Mitochondria, an organelle with independent genetic material in eukaryotic cells, is vital in cellular metabolism, apoptosis, diseases, aging, and other cellular processes. Due to its simple structure, compact arrangement, and low mutation rate, the exploration of the mitochondrial genome has been widely applied in molecular systematics and phylogeography studies [5, 6]. Compared to chloroplast DNA (cpDNA), plant mitochondrial DNA (mtDNA) is a complex and dynamic structure [6, 7]. It contains multiple repeat sequences in the non-coding regions, which can cause various recombinant sub-genomic forms of mtDNA [8, 9]. Therefore, the plant mtDNA map typically represents a circular DNA molecule composed of the entire mtDNA sequence, also known as the master chromosome [10]. Several studies have also independently reported existing subgenomic DNA molecules within plant mtDNA maps [8, 11, 12]. Although most studies have represented mtDNA structure as circular, linear structure has also been reported in some plants. For instance, maize S-type cytoplasmic male sterility (CMS) mtDNA primarily exists as multi-linear molecules [13], whereas the maintainer line mtDNA in the soybean-trisomic hybrid system exists in linear and circular structures simultaneously [12]. The plant mtDNA structure complexity is primarily attributed to the presence of various lengths of forward and reverse repeat sequences [14, 15]. The long repeat sequences (≥ 500 bp) cause frequent homologous recombination, transforming mtDNA into nearly equimolar mixtures, including interchangeable isomeric rings and subgenomic circles. In some plant species, such as *Dichanthium annulatum* [16], cucumber [8], and sugarcane [11], mtDNA was found to be composed of two or more independent circular DNA molecules, possibly due to shorter repeat sequence-caused abnormal recombination.

In addition to providing rich molecular data, the study of sweet persimmons' mitochondrial genome is conducive to exploring genetic evolution in the *Diospyros* genus. However, the availability of mitochondrial genome data of *Diospyros* species is far from the known number of species, and their mtDNAs are primarily identified in small fragments, resulting in a shortage of molecular information. Currently, *Diospyros oleifera* mtDNA is the only publicly available complete mitochondrial genome belonging to the *Diospyros* [17], which limits the in-depth molecular research on *Diospyros* species. The current study analyzed the mitochondrial genome of 'Taishuu' for the first time through second-generation and third-generation sequencing technologies, revealing its mitochondrial genome structural characteristics. Further, comparative genome analysis and phylogenetic tree construction were performed to evaluate the evolutionary relationships. These results can provide a foundation for studying the systematics and population evolution of 'Taishuu' and present molecular data for taxonomic research of the *Diospyros*.

## Results and analysis

### Features of mitochondrial genome

**Fig. 1:**
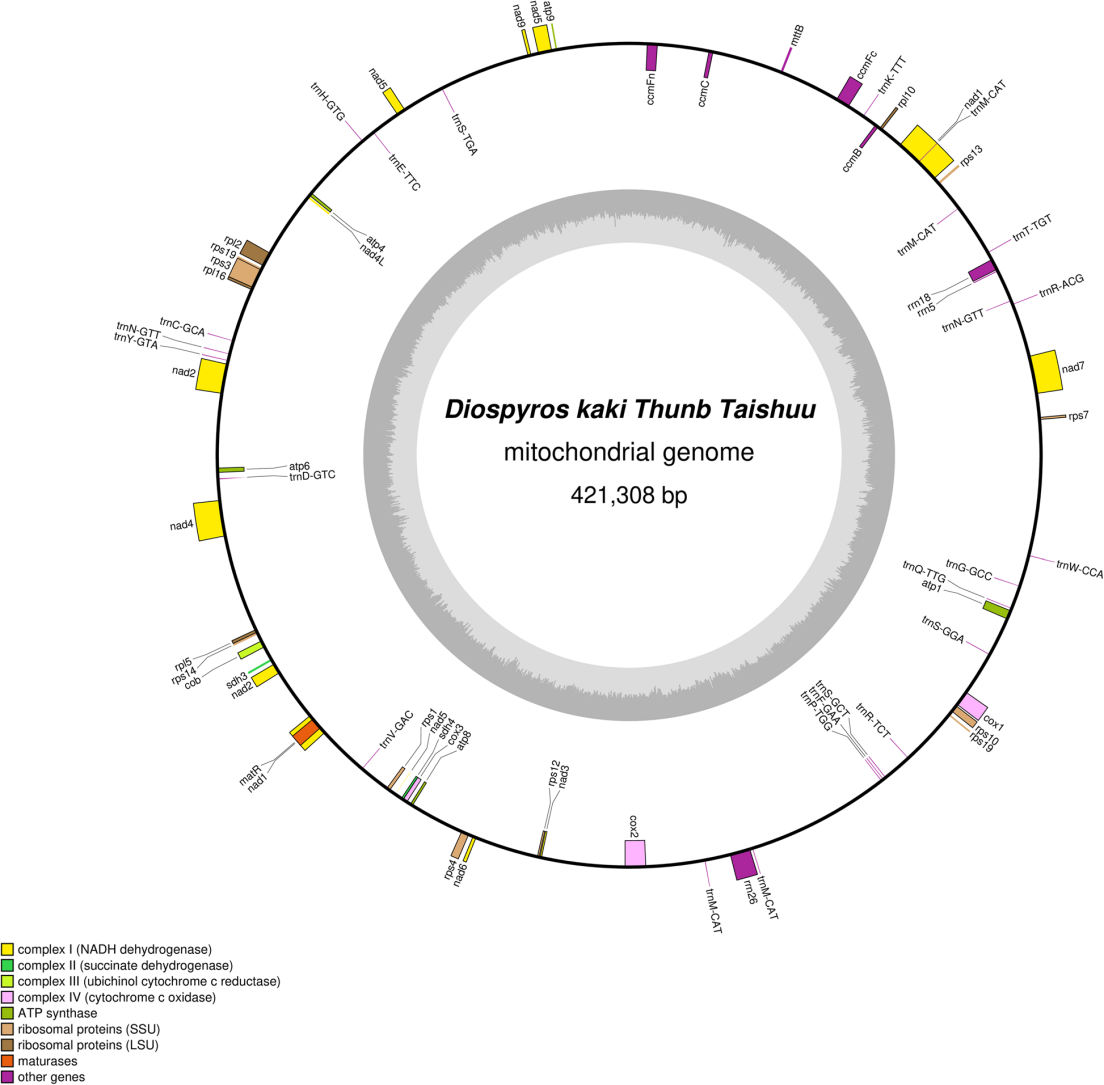
'Taishuu' Mitochondrial Genome Map. Genes encoded in the forward direction are located outside the circle, whereas those encoded in reverse are located inside. The inner gray circle represents GC content. In the linear presentation, genes encoded in the forward direction are above the circle, while those in the reverse are below

The second-generation and third-generation sequencing datas information can be found in Tab. S1. The depth of Sequencing datas is 370.38x. The 'Taishuu' persimmon mitochondrial genome sequence has been deposited to NCBI, with the accession number OR387071. The 'Taishuu' mt genome is a closed circular DNA molecule of 421,308 bp, with a 45.79% GC content. It comprises a total of 67 genes, including 24 tRNAs, 40 mRNAs, and 3 rRNAs. The 'Taishuu' persimmon mitochondrial genome encodes 40 proteins, which were classified into 10 categories, including ATP synthase (5), Cytochrome c biogenesis (4), Ubiquinol cytochrome c reductase (1), Cytochrome c oxidase (3), Maturases (1), Transport membrane protein (1), NADH dehydrogenase (9), Ribosomal proteins (LSU) (4), Ribosomal proteins (SSU) (10), and Succinate dehydrogenase (2) (Fig. 1, Tab. S2).

**Fig. 2:**
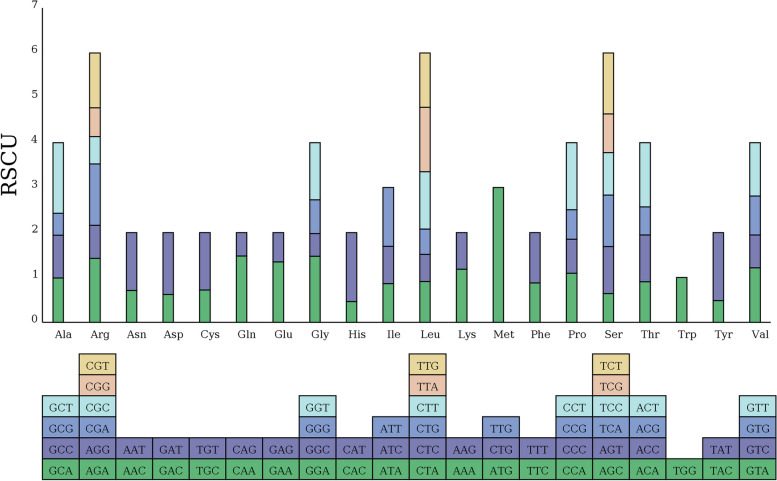
RSCU Bar Chart. The below squares represent all codons encoding each amino acid, while the height of the bars above represents the total RSCU value of all codons

The analysis of codon usage for the 'Taishuu' persimmon mitochondrial genome revealed that AGA, CCA, GGA, UGG, GUA, GCA, and CUA were the most prevalent codons for Arginine (Arg), Proline (Pro), Glycine (Gly), Tryptophan (Trp), Valine (Val), Alanine (Ala), and Leucine (Leu), respectively (Fig. 2). These findings explain the high proportion of AT nucleotides in the persimmon mitochondrial genome compared to GC.

The start codon for the protein-coding genes *cox1* and *nad4L* was ACG(ATG), whereas it was ATG for the remaining protein-coding genes. Four types of termination codons, including CGA, TAA, TAG, and TGA, were used at different frequencies of 4.76%, 45.24%, 16.67%, and 33.33%, respectively. TAA was the most frequently used stop codon (Tab. S2).

Previous studies suggested that the mt genomes of most terrestrial plants contain three rRNA genes [18]. The three rRNA genes, rrn18, rrn26, and rrn5, were annotated in the 'Taishuu' persimmon's mitochondrial genome of 1903 bp, 3382 bp, and 121 bp, respectively. In addition, 24 different tRNAs were identified in the 'Taishuu' mt genome, transporting 17 different amino acids. These findings explain that the same amino acid can be transported to different codons by two or more tRNAs (Tab. S2).

In total, 23 introns were detected in 14 genes (Tab. S3), including four introns in *nad1*, *nad2*, *nad5*, *nad7*, two in *cox2* and *nad4*, and one intron in *ccmFc*, *cox1*, *rps1*, *rps10*, *rps3*, *rpl2*, *trnR-TCT*, and *trnT-TGT*.

**Fig. 3:**
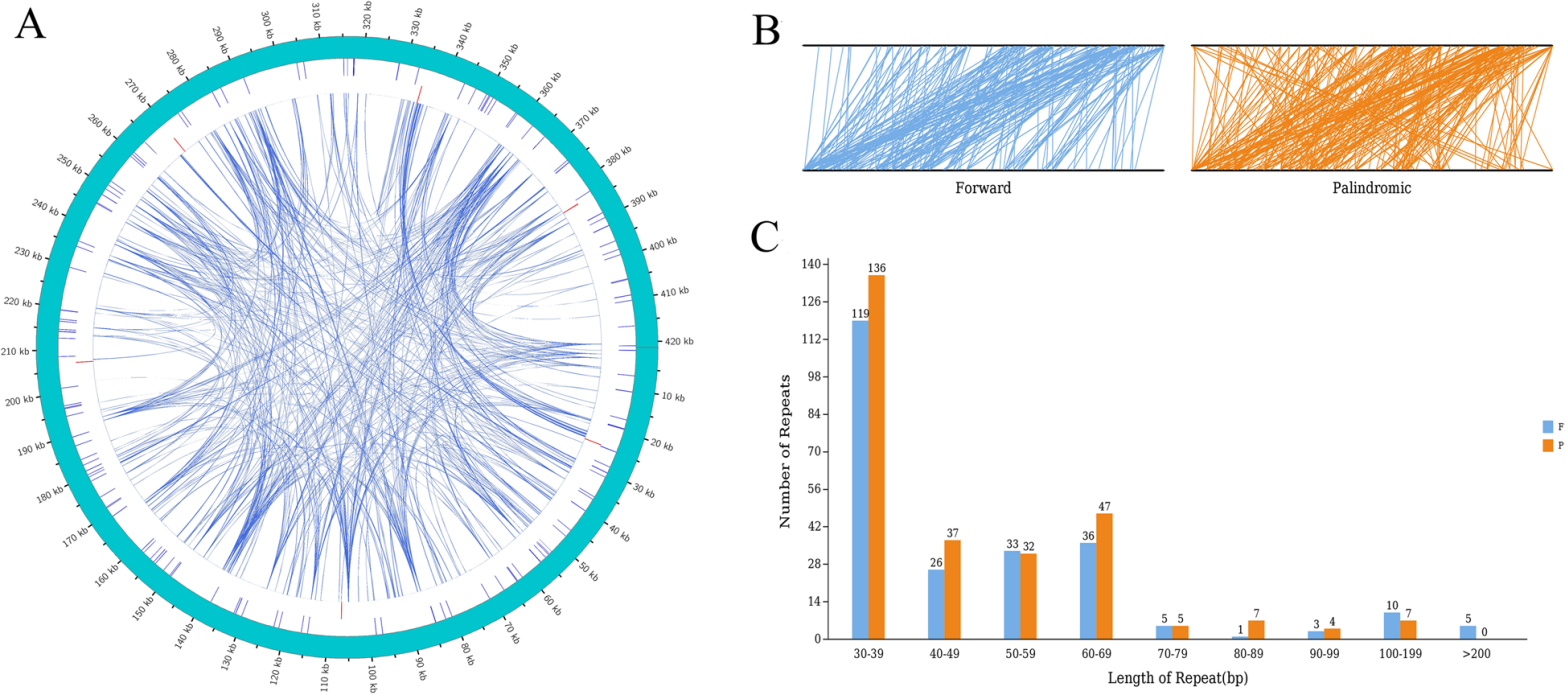
Repeat sequences in the *Diospyros kaki* Thunb. 'Taishuu' mitochondrial genome. **A** Distribution of repeat sequences on the mitochondrial genomic map. Note: The outermost circle represents simple repeat sequences, followed by tandem repeats, and lines in the innermost circle represent dispersed repeats. **B** Four types of dispersed repeats are distributed across the entire genome. The two black lines represent the mt genome, and identical repeat sequences are associated with the line segments. **C** Length distribution of interspersed repeats in the mitochondrial genome. The x-axis represents the types of interspersed repeats, and the y-axis represents the number of dispersed repeats

In the 'Taishuu' mitochondrial genome, 645 repeat sequences with lengths equal to or greater than 30 bp were identified, including 125 SSRs (Tab. S4), 7 tandem repeats (Tab. S5), and 513 dispersed repeats (Tab. S6). Detected SSR loci included mono-, di-, tri-, tetra-, penta-, and hexanucleotide repeats, with the three most common being A/T (25.6%), AG/CT (16.8%), and AAAG/CTTT (10.4%). The distribution of these repeat sequences on the genomic map is shown in Fig. 3A. Interspersed repeats (IRs) were dispersed across the genome; among these, 238 were direct repeats, whereas 275 were palindromic repeats (Fig. 3B). The longest direct repeat was 463 bp, and the longest palindromic repeat was 194 bp. The length distribution for direct and palindromic repeats is shown in Fig. 3C. Both types of repeats were most abundant within the 30 ~ 39 bp range.

## Comparative analysis of mitochondrial genomes

**Fig. 4:**
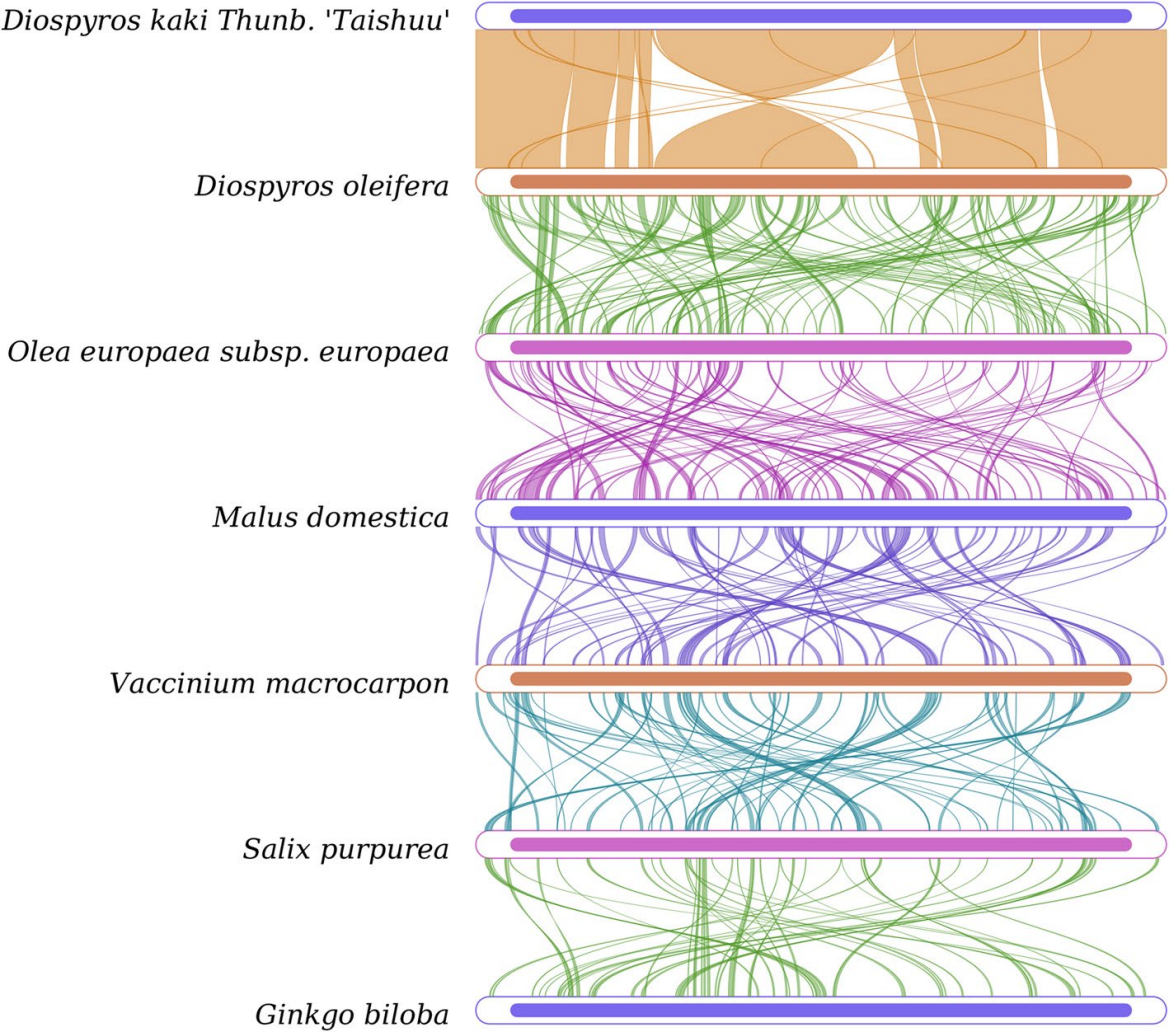
Collinearity Analysis of Mitochondrial Sequences. Each box in a row represents a genome, and the lines in between represent homologous regions

To further determine the gene rearrangements between 'Taishuu' persimmon and closely related varieties, a collinearity analysis was performed using the mitochondrial gene sequences of six closely related plant species available in NCBI (Fig. 4). The results showed that 'Taishuu' shared the closest relationship with *Diospyros oleifera*. Homologous gene fragments common in these species were relatively conserved and occupied most of the mitochondrial genome regions. The other homologous gene clusters might have undergone varying degrees of rearrangement or loss, reflecting that the mitochondria genomes of these species exhibit a general conservation trend with local dynamic changes during evolution. Based on the annotation results, we observed that the conserved regions primarily included protein-coding genes, while the variable regions primarily included intergenic regions. It can be speculated that the insertion or loss of unknown sequences in the intergenic region during the evolution of the mitochondrial genome might have led to rearrangement.

**Fig. 5:**
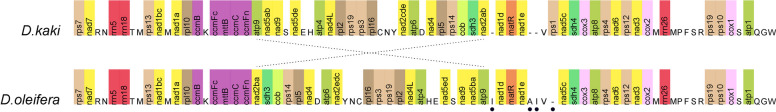
Schematic representation of the order of mtDNA genes In *Diospyros kaki* and *Diospyros oleifera*

Next, the mitochondrial gene sequences were compared among *Diospyros* species, using the *rps7* gene as the starting point. The arrangement of genes in the mitochondrial genomes of two *Diospyros* species is shown in Fig. 5. The arrangement of protein-coding genes in 'Taishuu' and *Diospyros oleifera* was consistent, with minor differences, whereas *nad2ab* and *atp9* were inverted. Minor gene inversion losses were observed, leading to differences in gene numbers.

## Evolutionary and phylogenetic analysis of mitochondrial protein-coding genes

**Fig. 6:**
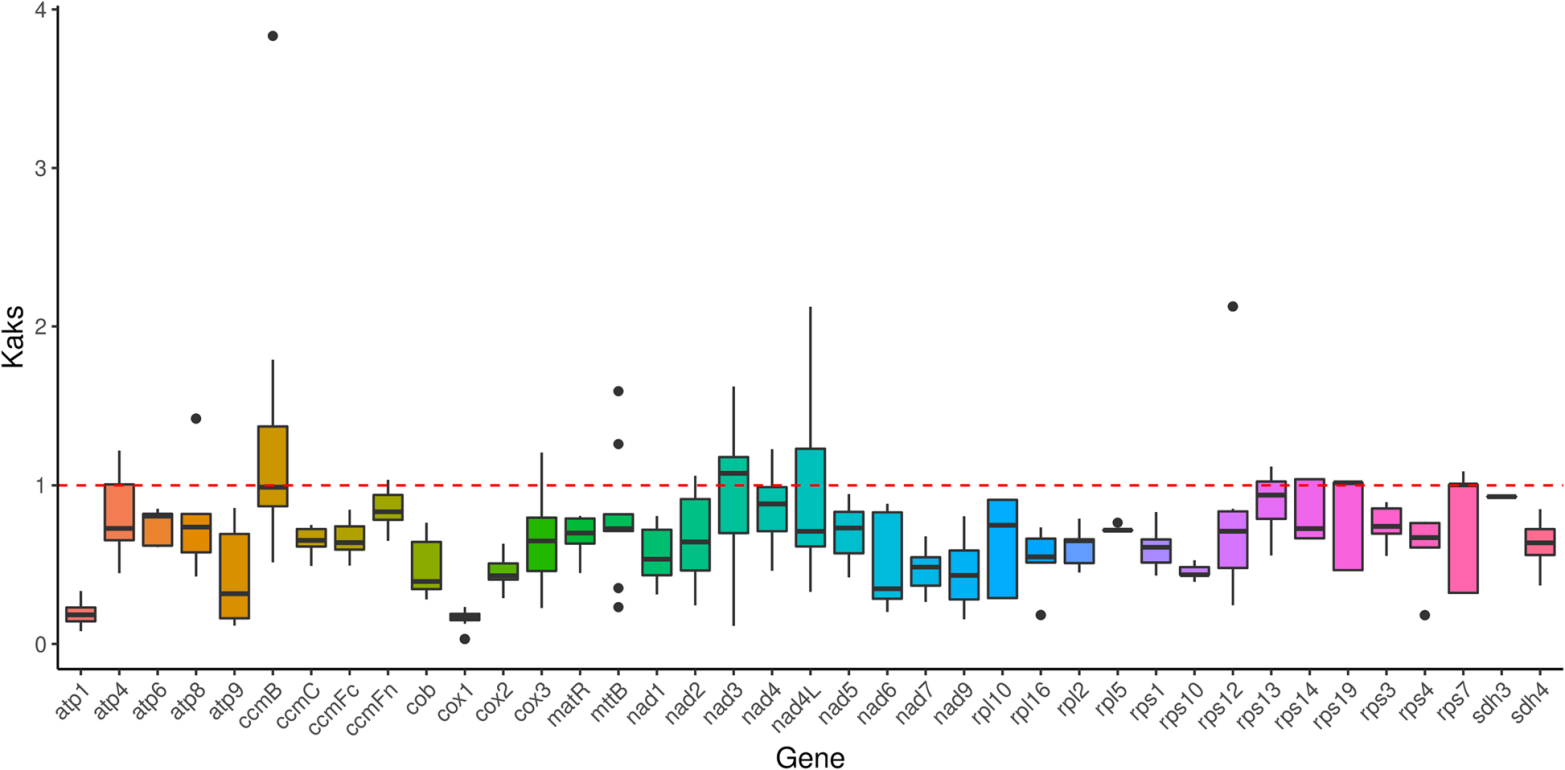
KaKs Box Plot Analysis Across Species. The horizontal axis represents gene names, and the vertical axis represents Ka/Ks values. In the box plot, the upper and lower endpoints of the vertical line within the rectangle represent the upper and lower boundaries of the data, respectively. The thick line inside the rectangle represents the median, the upper and lower edges represent the upper and lower quartiles, respectively, and the dots beyond the upper and lower boundaries of the data represent outliers

The amino acid changes caused by base mutation are known as nonsynonymous; otherwise, it is termed synonymous mutation. Nonsynonymous mutations are generally subjected to natural selection. The ratio of the nonsynonymous mutation rate (Ka) to the synonymous mutation rate (Ks) indicates the type of selection effect. A Ka/Ks ratio greater than 1 indicates a positive selection effect, whereas a ratio less than 1 suggests a purifying selection effect. We aligned 38 protein-coding genes in the 'Taishuu' mitochondrial genome with the mt genomes of six other species and analyzed them using Ka/Ks values (Tab. S7). As shown in Fig. 6, 35 genes were negatively selected during evolution, indicating that most of the protein-coding genes in the mt genome are relatively conserved. The Ka/Ks values of the protein-coding genes *ccmB* and *nad4L* were 1.26 and 1.07, respectively (greater than 1), indicating that these two genes might have undergone positive selection.

**Fig. 7:**
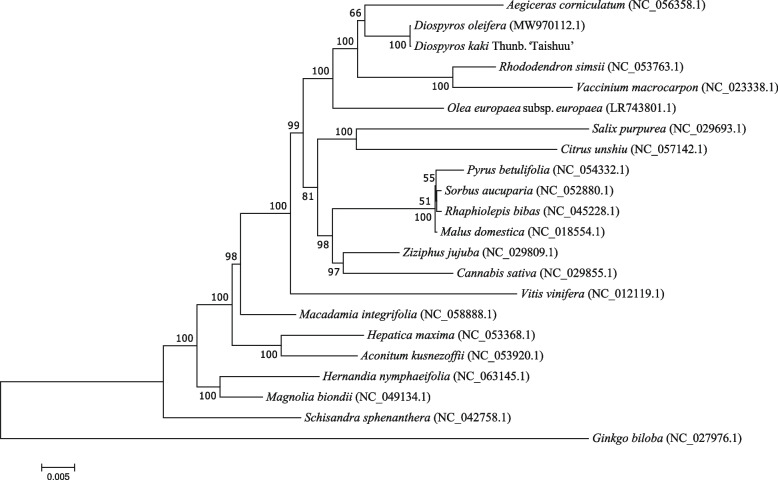
Mitochondrial Evolutionary Analysis. Evolutionary branch length, also known as genetic variation or evolutionary distance, represents the degree of branch variation; the shorter branch length represents a smaller difference and closer evolutionary distance. Distance scale: unit length of numerical differences between organisms or sequences, equivalent to the scale of the evolutionary tree. Bootstrap value is marked at the node position and used to evaluate the credibility of the branch

Phylogenetic analysis based on mitochondrial genes showed (Fig. 7) that 'Taishuu' and *D. oleifera*, two species from the *Ebenaceae* family, were clustered together, indicating their closer evolutionary relationship. 'Taishuu' was also closely located with *Aegiceras corniculatum* and *Rhododendron simsii*, belonging to the order *Ericales*, suggesting that mitochondrial protein-coding genes are desirable materials to unravel phylogenetic relationships among different plant species.

## Chloroplast and mitochondrial homologous sequence analysis

**Fig. 8:**
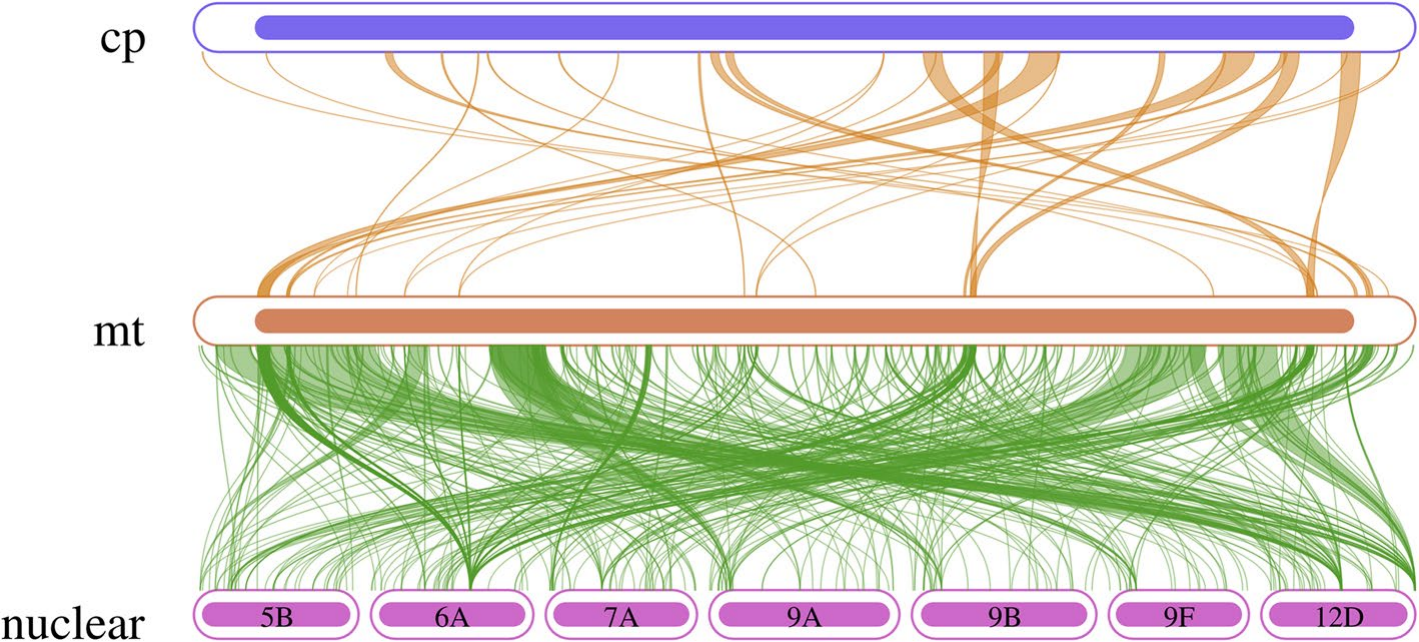
Homologous fragment analysis of mitochondrial genome, chloroplast genome, and nuclear genome. Chloroplast indicates chloroplast sequences, while the others indicate mitochondrial sequences. The genes from the same complex are marked with the same color, and the connecting line indicates homologous sequences

The blast was used to search for homologous sequences between chloroplasts and mitochondria, with an E-value of 1e-5 and a similarity equal to or more than 70%. The statistics are shown in Fig. 8. We screened 28 homologous fragments with a total length of 24,075 bp, accounting for 5.71% of the mt genome (Tab. S7). These homologous fragments contained eight annotated genes, including six tRNA genes: trnV GAC, trnW CCA, trnN GUU, trnD GUC, trnM CAU, and trnI UAU, as well as two other genes: rRNA genes (rrn18) and cytochrome c biogenesis genes (ccmC).

We screened 23 homologous genes between chloroplasts and nuclei (Tab. S9), which were distributed on 7 chromosomes (Fig. 8). They are atp9, ccmB, three NADH dehydrogenases (nad1,nad5, nad7), four Ribosomal proteins (rpl10,rps13, rrn26, rrn5) and fourteen Transfer RNAs (trnD-GTC, trnE-TTC, trnF-GAA, trnH-GTG, trnM-CAT, trnN-GTT, trnP-TGG, trnR-ACG, trnR-TCT, trnS-GCT, trnS-GGA, trnT-TGT, trnV-GAC, trnW-CCA). Mitochondria, chloroplasts, and nuclei share two homologous genes, which are trnV-GAC and trnW-CCA.

## Discussion

In the current study, the second and third-generation sequencing technologies were utilized to assemble the 'Taishuu' persimmon mitochondrial genome for the first time. The mitochondrial genome of 'Taishuu' persimmon was found to be circular, consistent with the previous reports that most plant mitochondrial genomes are circular [18]. The size of of 'Taishuu' persimmon mitochondrial genome is 421,308 bp with 45.79% GC content, slightly higher than that of *Oryza sativa* (43.8%) [19], *Zea mays* (43.9%) [20], *Hibiscus cannabinus* (44.9%) [21], and *Gossypium raimondii* (44.95%) [22] and equivalent to *Amborella trichopoda* (45.9%) [23]. This places it on the higher end regarding GC content among higher plants. Some genes contain one or more introns, possibly critical in gene expression regulation. Most terrestrial plants contain three rRNA genes [18]. These three rRNA genes, rrn18, rrn26, and rrn5, were annotated in the mitochondrial genome of 'Taishuu' persimmon.

In the *Diospyros* mitochondrial genome, the protein-coding region only accounted for 7.82% of the total length, whereas non-coding regions accounted for over 92%. The functional classification of protein-coding genes in the *Diospyros* mitochondrial genome is similar. The coding region of the genome was more conserved than the non-coding region, primarily responsible for the differences in the *Diospyros* mitochondrial genome [24]. The intergenic regions of the mitochondrial genome mainly consisted of repeat sequences, homologous sequences from the chloroplast genome, and homologous sequences from the nuclear genome. The repeat sequences included tandem sequences. Short and long repeat sequences are widely found in the mitochondrial genome [25]. They are essential for molecular recombination of the mitochondrial genome and are usually considered the major contributor to differences in plant mitochondrial genomes [23]. Most protein-coding genes in the *Diospyros* genus mitochondrial genome were conserved during the long evolution process. The studies of the mitochondrial genome provide sufficient molecular marker sites for the systematic evolution of this genus. The only mitochondrial information for the *Diospyros* genus currently available on the NCBI website is for *Diospyros oleifera*. Our phylogenetic results showed that *D._kaki* and *D. oleifera* were localized in the same branch, consistent with the previous mitochondrial genome collinearity analysis, revealing a close evolutionary relationship.

Plant mitochondrial genomes always contain sequences transferred from chloroplast genomes, usually accounting for 1–12% of the total length [26]. Nearly one-third of tRNA genes originate from chloroplasts and have gradually migrated during evolution [27]. A previous study on higher plants has shown that approximately 42% of the chloroplast genome fragments have been integrated into the 773,279 bp grape (*Vitis vinifera*) mitochondrial genome, including more than thirty chloroplast protein-coding genes and 17 tRNA genes [28]. Furthermore, in the 982,833 bp zucchini (*Cucurbita pepo*) mitochondrial genome, more than 113 kb chloroplast genome fragments were identified [29], whereas the chloroplast sequences in rice accounted for 6.2% of the genome [19]. Our study identified 24075 bp of homologous sequences in the 'Taishuu' persimmon mitochondrial and chloroplast genomes, accounting for approximately 5.71% of the mitochondrial genome. These fragments may be critical during evolution.

DNA migration occurs slowly during evolution. During the transfer process, chloroplast genome fragment often carries some chloroplast protein-coding genes into the mitochondrial genome; however, these genes lose their integrity and become pseudogenes following incorporation into the mitochondrial genome, possibly due to genomic sequence recombination [30]. Our analysis of the 'Taishuu' persimmon mitochondrial genome for chloroplast migration sequences depicted the same conclusion. In contrast, the non-protein coding genes function normally after being transferred into the mitochondrial genome. Complete structures of 24 different tRNA genes were observed in 'Taishuu' persimmon, which might have normal transport functions, indicating that tRNA genes are more conserved than protein-coding genes in the mitochondrial genome. This characteristic may be unique to higher plant mitochondria during evolution. Currently, the mechanisms and expression patterns of sequence migration between 'Taishuu' persimmon genomes are unknown. Therefore, further perfection of the 'Taishuu' persimmon whole genome project in this study will help address these research gaps.

For the first time, we applied mitochondrial whole-genome sequences to the evolutionary analysis of 'Taishuu' persimmon. A phylogenetic relationship analysis was conducted based on the publicly available mitochondrial genome of one species from the *Ebenaceae* family and 20 other published plant mitochondrial genome sequences. The results showed clear taxonomic distinctions of each species. 'Taishuu' and *D. oleifera,* belonging to the *Ebenaceae* family forming one cluster. Nevertheless, consistent with the biological classification, they were also phylogenetically close to *Aegiceras corniculatum* and *Rhododendron simsii*, belonging to the order *Ericales*. The current study on the mitochondrial genome represents only the tip of the iceberg, and additional research must be carried out to obtain accurate conclusions concerning *Diospyros* genus mitochondrial genome. In addition, this report verifies that mitochondrial genome sequences have certain advantages in phylogenetic evaluation [18]. Our study provides new insights into the genetics, systematics, genomics, and evolution of *D. kaki* and the *Diospyros* genus.

## Experimental methods

### Plant material and sequencing

The leaves of *Diospyros kaki* Thunb. 'Taishuu' were collected from a farm at the Cotton Research Institute of Shanxi Agricultural University in Yuncheng City, Shanxi Province, China. Nanjing Genepioneer Biotechnologies Co., Ltd conducted sequencing.

A combination of second and third-generation sequencing strategies was employed in this study. The whole mitochondrial genome was subjected to second-generation sequencing using the Illumina Novaseq6000 platform and third-generation sequencing using the Oxford Nanopore PromethION sequencer. The raw third-generation sequencing data were aligned to the reference gene sequences (https://github.com/xul962464/plant_mt_ref_gene) using minimap2 (2.1), enabling the retrieval of the complete mitochondrial genome from third-generation sequencing. The Canu program was used for the correction of third-generation data, and bowtie2 was used to align the second-generation data to the corrected sequence. Finally, the Unicycler tool was applied with default parameters to assemble the aligned second-generation data and corrected third-generation data [31].

### Annotation of mitochondrial gene structure

The annotation process of mitochondrial genes was as follows: (1) Protein-coding genes and rRNA genes were annotated using blast against published reference plant mitochondrial sequences, followed by manual adjustment based on closely related species. (2) tRNA genes were annotated using tRNAscan-SE (http://lowelab.ucsc.edu/tRNAscan-SE/) [32]. (3) ORFs (Open Reading Frames) were annotated using ORF Finder (http://www.ncbi.nlm.nih.gov/gorf/gorf.html), with a minimum length of 102 bp. Redundant and overlapping sequences with known genes were excluded. Sequences longer than 300 bp were aligned to the nr database for annotation. A mitochondrial genome map was created using OGDRAW (https://chlorobox.mpimp-golm.mpg.de/OGDraw.html).

### Sequence characteristic analysis

The relative synonymous codon usage (RSCU) was calculated using a custom Perl script to filter unique coding sequences (CDS). It is well established that the repeat sequences include simple sequence repeats (SSR), tandem repeats, and dispersed repeats. In this study, SSR repeats were identified using MISA software (v1.0), tandem repeats were identified using TRF software (trf409.linux64), and dispersed repeats were identified using blastn (v2.10.1) (Version 2.10.1, parameters: word_size 7, evalue 1e-5, remove redundancy, eliminate tandem repeats). The identified repeats were visualized using Circos v0.69–5.

KaKs analysis was performed using mafft v7.310 (https://mafft.cbrc.jp/alignment/software/) to align the ‘Taishuu’ mitochondrial genome to the mitochondrial gene sequences of six other species. The KaKs values of genes were calculated using KaKs_Calculator v2.0 (https://sourceforge.net/projects/kakscalculator2/), with the MLWL calculation method [31]. The GenBank IDs for the mitochondrial gene sequences from six other sequences were as follows: *Diospyros oleifera*: MW970112.1, *Vaccinium macrocarpon*: NC_023338.1, *Malus domestica*: NC_018554.1, *Olea europaea* subsp. *Europaea*: LR743801.1, *Ginkgo biloba*: NC_027976.1, and *Salix purpurea*: NC_029693.1.

### Comparative analysis of mitochondrial genomes

Sequences of 'Taishuu' and 21 other species were aligned using the MAFFT software (v7.427). Select species based on the basic unit of biological classification by searching for publicly available mitochondrial genome information on the NCBI website. Firstly, select different persimmon species according to the same genus of persimmons; Secondly, select according to the same persimmon family but different persimmon genera; Thirdly, select according to the same order of persimmons but different families of persimmons, classify from the lowest to the highest level, and expand the selection range in sequence until the plant kingdom stops selecting. The information of 21 other varieties is provided in Tab. S10.

The maximum likelihood method-based evolutionary tree was constructed using CDS. The aligned sequences were concatenated, trimmed with trimAl (v1.4.rev15) software (parameter: -gt 0.7), and analyzed using j Model Test-2.1.10 software to predict the model, which was determined to be the GTR type. Next, the evolutionary tree was constructed using the RAxML v8.2.10 (https://cme.h-its.org/exelixis/software.html) software and the GTRGAMMA model, with a bootstrap value of 1000.

A comparative genomics analysis between the assembled sequence and the selected other mitochondrial sequences was performed using Blastn (2.10.1 +). Using parameters such as -word_size as 7 and E-value as 1e-5, aligned fragments longer than 300 bp were screened to draw the collinearity diagram.

## Chloroplast and mitochondrial homologous sequence analysis

The blast was performed to search the homologous sequences between mitochondria and *Diospyros kaki* chloroplast (GenBank number: NC_030789.1) and nuclear genome (GenBank number: GCA_028777405.1), with similarity set at 70% and E-value 1e-5. The visualization was performed using Circos v0.69–5.

### Supplementary Information

**Supplementary Material 1.**

**Supplementary Material 2.**

**Supplementary Material 3.**

**Supplementary Material 4.**

**Supplementary Material 5.**

**Supplementary Material 6.**

**Supplementary Material 7.**

**Supplementary Material 8.**

**Supplementary Material 9.**

**Supplementary Material 10.**

## Data Availability

The sequence and annotation of the *Diospyros kaki* mt genome were submitted to the NCBI. The accession number in Gene Banks is OR387071 (https://www.ncbi.nlm.nih.gov/nuccore/OR387071).

## Abbreviations

Ala: Alanine
Arg: Arginine
cpDNA: Chloroplast DNA
*D. kaki*: *Diospyros kaki*
*D. oleifera*: *Diospyros oleifera*
Gly: Glycine
Leu: Leucine
mtDNA: Mitochondrial DNA
ORFs: Open Reading Frames
PCA: Pollination-constant astringent
PCNA: Pollination constant & non-astringent
PVA: Pollination-variant astringent
PVNA: Pollination-variant non-astringent
Pro: Proline
Trp: Tryptophan
Val: Valine
