# Supplementary figures and images for: Mitochondrial genome features and systematic evolution of diospyros kaki thunb 'Taishuu'
Source: BMC Genomics. 2024 Mar 18;25:285. doi: 10.1186/s12864-024-10199-0 (PMC10946091; doi:10.1186/s12864-024-10199-0)

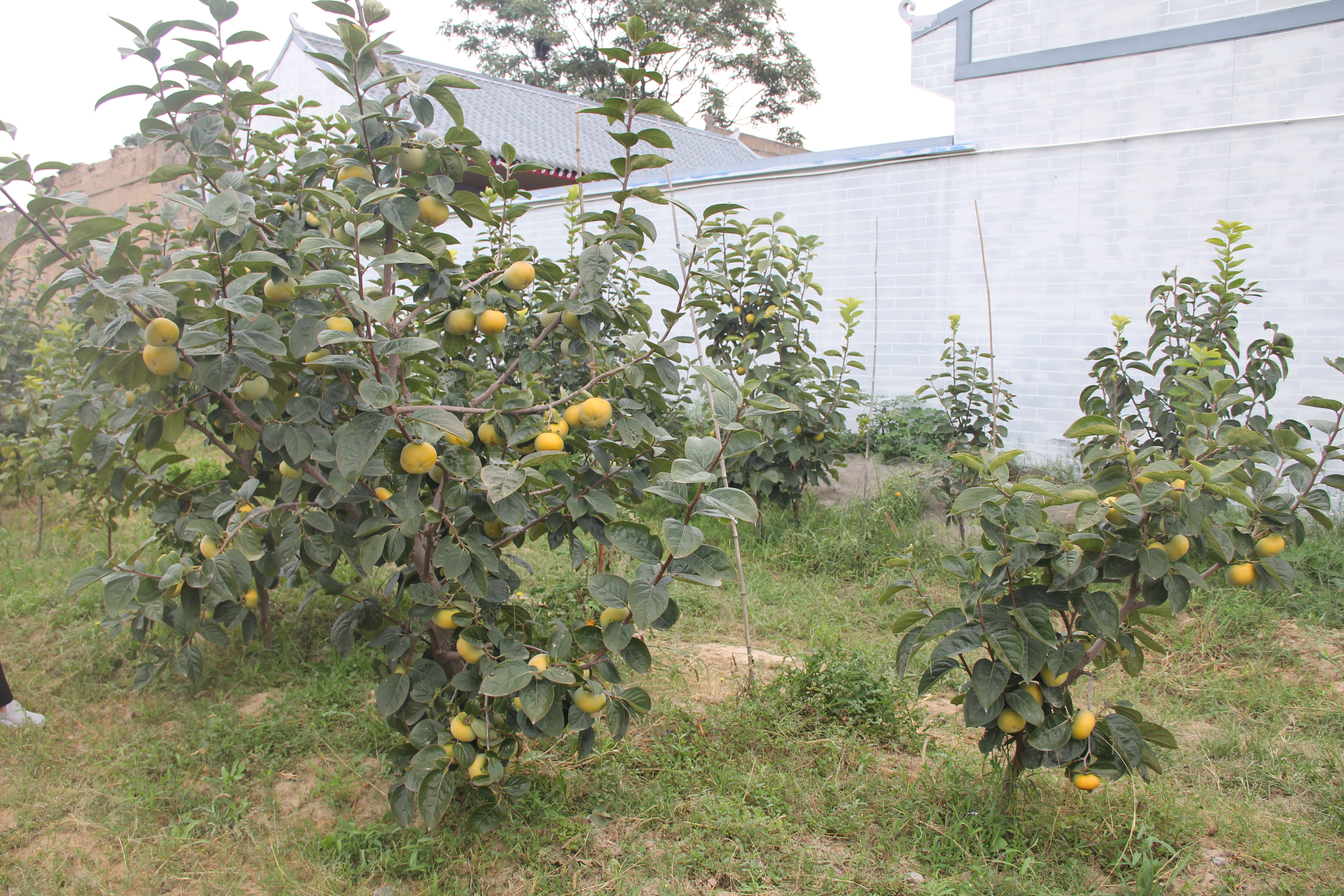

Supplement: Supplementary file 2 — Supplementary Material 2. [file 12864_2024_10199_MOESM2_ESM.jpg]
